# Giant Cell Tumors of the Axial Skeleton

**DOI:** 10.1155/2012/410973

**Published:** 2012-02-08

**Authors:** Maurice Balke, Marcel P. Henrichs, Georg Gosheger, Helmut Ahrens, Arne Streitbuerger, Michael Koehler, Viola Bullmann, Jendrik Hardes

**Affiliations:** ^1^Department of Trauma and Orthopedic Surgery, Cologne-Merheim Medical Center, WittenHerdecke University, Ostmerheimer Street, 200, 51109 Cologne, Germany; ^2^Department of Orthopedic Surgery, University of Münster, Albert-Schweitzer-Straße 33, 48149 Muenster, Germany; ^3^Department of Clinical Radiology, University of Münster, Albert-Schweitzer-Straße 33, 48149 Muenster, Germany

## Abstract

*Background*. We report on 19 cases of giant cell tumor of bone (GCT) affecting the spine or sacrum and evaluate the outcome of different treatment modalities. *Methods*. Nineteen patients with GCT of the spine (*n* = 6) or sacrum (*n* = 13) have been included in this study. The mean followup was 51.6 months. Ten sacral GCT were treated by intralesional procedures of which 4 also received embolization, and 3 with irradiation only. All spinal GCT were surgically treated. *Results*. Two (15.4%) patients with sacral and 4 (66.7%) with spinal tumors had a local recurrence, two of the letter developed pulmonary metastases. One local recurrence of the spine was successfully treated by serial arterial embolization, a procedure previously described only for sacral tumors. At last followup, 9 patients had no evidence of disease, 8 had stable disease, 1 had progressive disease, 1 died due to disease. Six patients had neurological deficits. *Conclusions*. GCT of the axial skeleton have a high local recurrence rate. Neurological deficits are common. En-bloc spondylectomy combined with embolization is the treatment of choice. In case of inoperability, serial arterial embolization seems to be an alternative not only for sacral but also for spinal tumors.

## 1. Introduction

Giant cell tumor of bone (GCT) is a rare skeletal lesion that typically arises in the metaepiphyseal ends of long bones [1–3]. Its peak incidence is between 30 to 40 years of age. Although classified as benign it shows locally aggressive behavior [4–7].

The treatment is mainly surgical and consists of intralesional curettage of the tumor followed by bone cement packing or bone grafting of the defect. Depending on the surgical procedure the local recurrence rate significantly varies from approximately 10% to 40% and is the lowest if high-speed burring of the margins after curettage and bone cement packing is used [2, 8–11].

Whereas these treatments are nowadays well accepted for “typical” GCT, recommendations on treating tumors of rare localizations such as small bones, pelvis, spine, or sacrum are still unclear [1–3, 12–18]. Especially tumors of the axial skeleton, mainly spine and sacrum, seem to be particularly complicated to treat. This is most likely due to the limited surgical accessibility and proximity to spinal cord and nerve roots. Possible treatments range from intralesional resection to en bloc spondylectomy with various adjuncts such as irradiation or arterial embolization [12, 15, 19–25].

The literature provides only small case series of spine or sacral GCT with mostly short follow-up periods [12, 15, 19–25]. In this report we add our experience with treatment of GCT affecting the axial skeleton, and discuss our results with respect to the current literature.

## 2. Materials and Methods

Nineteen patients with histologically certified benign GCT of the axial skeleton have been included in this study. They were collected from the GCT database of the corresponding author that includes 282 patients since 1980. The stored information was received from patient records, surgical protocols, and histological and radiological findings. The last followup was done via personal contact in the outpatient clinic of the senior author at a mean of 51 (15–133) months. 13 tumors were located in the sacrum, 6 tumors were located in the mobile spine: 4 thoracic and 2 lumbar. For an overview of the patient collective see Table 1.

### 2.1. Primary Treatment

**Figure 1:**
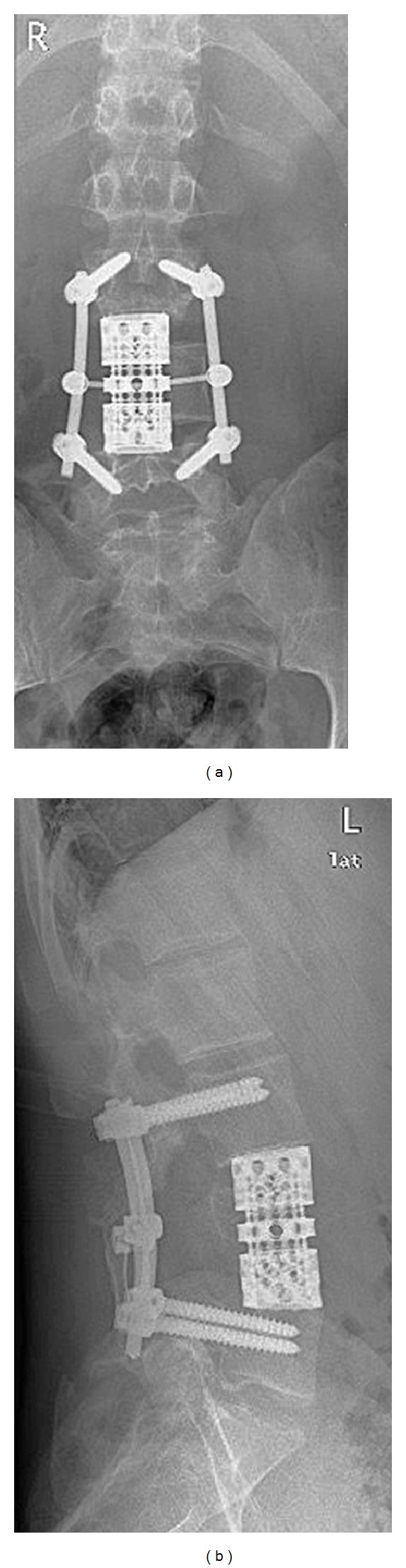
X-ray after dorsoventral spondylectomy. Postoperative X-ray of case 6. Dorsoventral intralesional spondylectomy of L4, dorsal instrumentation from L3 to L5, and titanium cage interposition with bone graft.

For detailed information see Table 1. All 6 patients with GCT of the mobile spine (cases 1–6) were primarily surgically treated by intralesional procedures after preoperative embolizasion. One patient (case 1) received a partial resection through a ventral approach, filling of the defect with bone graft and ventral stabilization. One patient (case 2) was treated through a dorsal approach by intralesional curettage, burring of the margins, bone grafting plus bone cement packing, and dorsal stabilization. Two thoracic tumors (cases 3 and 4) were treated by dorsoventral procedures with dorsal instrumentation and stabilization followed by intralesional (due to spinal cord displacement) tumor resection. Both lumbar tumors (cases 5 and 6) were treated by dorsoventral spinal body resection and reconstruction with a vertebral body replacement filled with autologue bone graft (Figure 1). Due to the soft tissue component with invasion of the right psoas muscle and displacement of the spinal cord, both resections were considered as intralesional.

**Figure 2:**
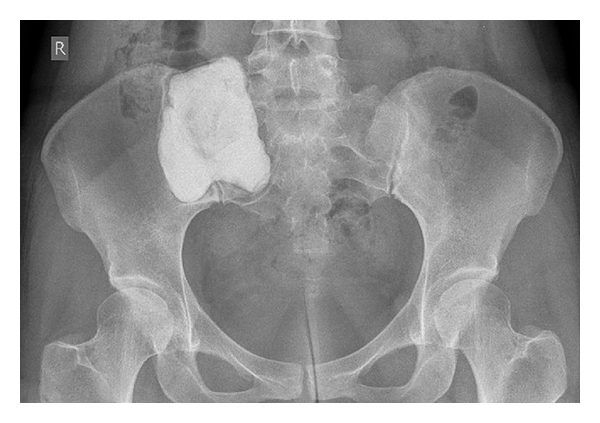
X-ray after curettage and bone cement packing of a sacral tumor. Postoperative X-ray of case 14 after intralesional curettage and bone cement packing.

 The patients with sacral tumors (cases 7–19) were either treated primarily surgically (cases 7–16) or with external beam irradiation (EBI) only (cases 17–19). Five patients (cases 7–11) were treated by intralesional curettage and bone cement packing (Figure 2). One patient (case 12) received partial resection, mainly of the soft tissue component, followed by chemotherapy and EBI. Four patients (cases 13–16) received preoperative selective arterial embolization (SAE) followed by intralesional curettage and bone graft (case 13) or curettage and bone cement packing (case 14), in 2 cases combined with postoperative EBI (cases 15-16). Cases 15 and 16 presented with spinal cord compression, thus only partial removal of the tumor was possible. Three patients (cases 17–19) were not operated on, but were solely treated by EBI (cases 17 and 18) which in one case was followed by SAE three months later (case 19).

## 3. Results

The mean age at first diagnosis of all patients was 27.4 (range 17 to 61) years. Patients with affection of the sacrum were slightly older (mean 29.2 years) than the spine patients (mean 23.5 years). 13 patients were female, 6 were male. Three out of six spinal patients (cases 3, 5, 6) and two out of thirteen sacral patients (cases 16 and 19) had neurological deficits at initial presentation (Table 1).

Concerning the radiological findings according to Campanacci and Enneking [2, 26] all 15 patients for whom the respective information was available presented with stage III lesions. The same 15 patients presented with a soft tissue component.

### 3.1. Local Recurrences

The mean period from primary treatment to detection of the recurrence was 11.3 (7–21) months; 6 of the 7 recurrences developed within the first year (see Table 1). All patients were seen every 3 month for the first two years postoperatively and evaluated by X-rays. If there were any suspicious findings or a worsening of symptoms additional diagnostics such as MRI or CT were performed. Until the 5th postoperative year patients were seen every 6 months and from then on once a year.

**Figure 3:**
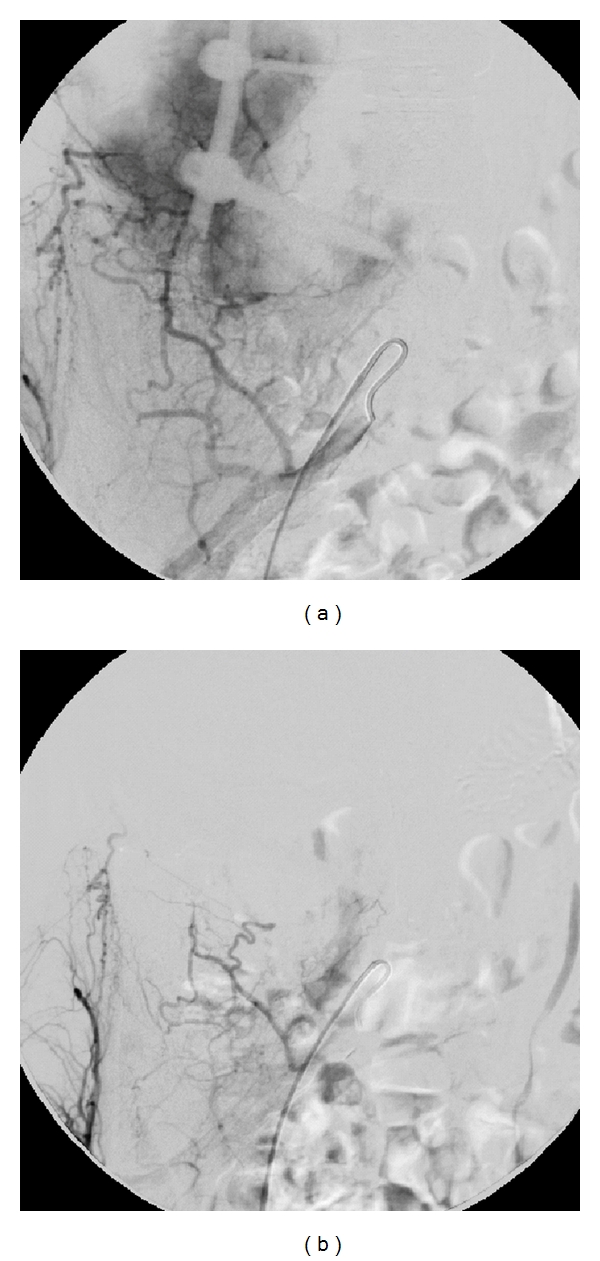
Angiogram before and after selective arterial embolization. (a) Preembolization angiogram of the right internal iliac artery demonstrating a massive hypervascularization of the giant cell tumor (case 6). (b) After embolization with embozene microspheres (250 *μ*m) a complete devascularization of the giant cell tumor was achieved.

Four of the spine cases developed a local recurrence (cases 1, 3, 4, 6) of which one case (case 4) developed two recurrences. Case 1 was initially treated by intralesional tumor resection and bone grafting through a ventral approach. The recurrence was successfully treated by another intralesional tumor resection and bone grafting, again through a ventral approach. He developed a loss of sensory function and paresis at Th 9 level, with a persistent footdrop right after surgery. Cases 3 and 4 were initially treated by partial intralesional vertebral body resection, bone grafting, and dorsal stabilization through a combined dorsoventral approach. The recurrence of case 3 was treated by intralesional tumor resection, decompression of the spinal cord, and elongation of the internal fixation through a dorsal approach. She developed pulmonary metastases two month later and additionally received chemotherapy and irradiation (see below) resulting in stable disease. The recurrence of case 4 was treated by intralesional tumor resection through a ventral approach but developed another recurrence six months later. She was then treated by intralesional retroperitoneal partial tumor resection and bone cement packing. Intraoperatively she had a severe blood loss due to laceration of the aorta. She was scheduled for EBI but eventually died 13 days after the last operation due to respiratory failure. Case 6 was primarily treated by dorsoventral intralesional spondylectomy, dorsal stabilization, and cage implantation with bone grafting. Due to infiltration of the psoas muscle and invasion of the spinal canal this resection was still considered intralesional. He presented with a huge local recurrence surrounding the abdominal aorta causing sensitive disorders. He was considered as inoperable and successfully treated by a series of SAE directly after detection of the recurrence, 1 months and 6 months later. Each SAE was done until complete devascularization of the tumor vessels was achieved (Figure 3). At last followup he presented with a stable disease without neurological impairments but still needing daily pain medications. Since the recurrence, he additionally has been receiving oral bisphosphonates (clodronate 800 mg) twice a day.

Three of the sacrum cases developed a local recurrence (cases 7, 9, 17). The first two were primarily treated by intralesional curettage and bone cement packing, the last by EBI only. Case 7 developed a local infection 1 month after initial surgery that was cured by repeated curettage and bone cement packing in combination with systemic antibiotics. After this second operation he suffered from irritation of the left S1 nerve root resulting in neurologic claw toes. He presented with a soft tissue recurrence without affection of the bone and was successfully treated by resection of the respective tissue. The recurrence of case 9 was treated by preoperative SAE and intralesional partial resection but was progressive 6 months later and again treated by SAE. Another 6 months later the tumor was again progressive and was treated by EBI. At last follow up the patient showed incomplete paralysis of the flexors and extensors of the left foot and the tumor was still progressive. At the time of writing of this report he was scheduled for experimental denosumab (human RANK ligand antibody) treatment. Case 17 showed a tumor regression after the first EBI but presented with a progress 21 months after initial treatment. He received another EBI combined with oral bisphosphonates (clodronate). At the last follow up he had stable disease, was not in pain but still taking bisphosphonates.

### 3.2. Pulmonary Metastases

For the detection of pulmonary metastases patients alternately received chest X-rays and CT scans of the chest every 6 months for the first two years. In case of suspicious findings or local recurrence a chest CT scan was performed. Two patients with affection of the spine (cases 3 and 5) developed pulmonary metastases 13 and 16 months after primary treatment: case 3 two months after local recurrence, case 5 without signs of recurrence. Both were initially treated by dorsoventral (partial) vertebral body resection and dorsal instrumentation. When the metastases of case 3 were detected she also showed a local progress with destruction of Th 7 to Th 9, tumor tissue surrounding the thoracic aorta, displacement of the cava and heart, and pleural and pericardial effusion. She was treated by incomplete resection of the metastases followed by EBI and 4 cycles of chemotherapy (ifosfamid/cisplatin). At last followup 70 months after the metastases she was doing well, had stable metastases and local findings and was pregnant. Case 5 presented with unspecific findings in the chest CT since first diagnosis. 16 months later the pulmonary findings, were progressive and were confirmed to be metastases after surgical resection. At last followup she had no evidence of disease, was doing well and taking oral bisphosphonates (clodronate) since the last surgery.

### 3.3. Clinical Outcome

At last followup 9 patients (3 spine, 6 sacrum) had no evidence of disease, 8 (2 spine, 6 sacrum) had stable disease, 1 (sacrum) progressive disease, and 1 (spine) died due to disease. Besides the complications mentioned above two patients (case 8 and 12) developed a local infection after curettage of a sacral tumor and were successfully treated by systemic antibiotics and repeated surgical revisions.

Concerning the spine three patients were free of complaints, one (case 6) was on daily pain medication and one (case 1) had neurological impairments. Concerning the sacrum, 8 patients were free of complaints, and five (cases 7, 9, 11, 16, 19) had neurological impairments (Table 1).

## 4. Discussion

In giant cell tumor of bone affection of the axial skeleton is extremely rare. From our database comprising 282 patients only 6.7% occurred in the spine or sacrum. Thus they are even less frequent than GCT of the pelvis with 8.7% of our patients. This is in accordance with previous reports [19, 22, 25]. It is known that GCT slightly prefer females with a ratio around 1.2 to 1 [1, 4, 6, 7, 27]. For the axial skeleton 13 of our 19 patients (68.4%) were female which is a much higher rate and in contrast to most previous reports [19, 22]. Sanjay published a comparable gender predilection for GCT of the spine [25]. Our patients with a tumor of the mobile spine were younger than sacrum patients or patients with tumors of the long bones, which is in contrast to previous reports [19, 25]. Due to the small amount of patients this might be incidental. In the study by Martin and McCarthy [19] comprising 23 patients of the spine and sacrum the minority of 10 patients affected the sacrum. In contrast to this in our study affection of the sacrum was more frequent than of the mobile spine. In the publication by Sanjay et al. from 1993 about 24 patients with GCT of the spine treated at the Mayo Clinic [25] affection of the cervical, thoracic, and lumbar regions was equally distributed. In our patient collective as in the case series by Ma et al. from 1987 GCT only occurred in the thoracic and lumbar spines [28]. The local recurrence rates of 66.7% (4 of 6) for the mobile spine and 15.4% (2 of 13) for the sacrum are significantly higher than that of GCT of the long bones or pelvis [1, 18, 29]. This is in accordance with previous reports of Martin and McCarthy who published a recurrence rate of 22% for sacral and 31% for spinal GCT [19] and Sanjay et al. who published a rate of 41.7% for spinal GCT [25]. Especially for intralesional resections or tumors that have been solely treated by irradiation it is a matter of definition to differentiate between recurrence or progress of left-over tumor tissue. However, the statement of Martin and McCarthy [19] that GCT of the axial skeleton, especially of the spine, carry a much worse prognosis, can be affirmed.

The rate of grade III tumors according to Campanacci and Enneking [2, 26] as well as the rate of soft tissue extension is higher than in patients with typical GCT [1, 2, 6, 30, 31]. The diagnosis might be delayed as the first symptom back pain is extremely frequent in the orthopaedic practice and can easily be misinterpreted. On the other hand it might be assumed that the tumors of the spine and sacrum are more aggressive.

As described for GCT of the long bones, tumors of the axial skeleton are also capable of producing benign pulmonary metastases. In our collective this occurred in two of six patients with affection of the spine and in none of the sacrum cases. Whereas for typical GCT the metastasis rate of less than 2% [1, 29] is extremely low it seems to be higher for spinal tumors with published rates of up to 13.7% [24]. In contrast to this Martin and McCarthy did not find any metastases in 10 sacral and 13 spinal GCT [19].

Treatment strategies for typical GCT of the long bones are generally well defined. Treatment of choice is intralesional curettage, burring of the cavity, and packing with bone cement. This procedure in the majority of cases leads to a good functional outcome with a local recurrence rate of around 15% [1, 29, 31, 32]. However, this strategy can hardly be transferred to tumors of the spine or sacrum. These tumors often infiltrate the spinal canal, compressing the spinal cord. Thus complete curettage is hardly possible and adjuncts such as bone cement, phenol, or cryotherapy cannot be used. Therefore in three sacral cases we decided not to offer surgery (case 17–19). All three presented with large tumors very close to the neural structures. Surgical removal would have resulted in severe neural damage. Nowadays we would preferably perform SAE instead of primary irradiation whenever surgery is not reasonable. Local recurrences of the spine are much more difficult to treat than of the extremities. Additionally, tumors of the axial skeleton seem to be diagnosed at a later stage of disease often presenting with a soft tissue mass. Wide or marginal excision of the tumor or en bloc resections may result in a lower recurrence rate but often cause unacceptable neurological impairments [19]. Liljenqvist et al. stated in a publication on malignant tumors of the spine that en bloc spondylectomy enables wide or marginal resection in most cases with acceptable morbidity [33].

Four of our six spine patients had a local recurrence. Although one of them received spondylectomy, the margins were defined as intralesional in all cases due to soft tissue expansion and compression of the spinal cord or nerve roots. This might explain the recurrence also after spondylectomy, which is generally lower than after partial resections [19, 23, 25]. After review of the literature we also recommend en-bloc resection whenever possible as this results in the lowest recurrence rates even if not confirmed by our results. We nowadays aim for at least marginal en bloc spondylectomy whenever possible.

The recurrence is always more difficult to treat than the primary tumor, thus its occurrence should be avoided as much as possible. Serial SAE seems to be a treatment option even if the tumor affects the mobile spine. This procedure was originally described for sacral tumors by Lin et al. in 2002 [34]. He treated 18 patients with GCT of the sacrum with a series of selective intra-arterial embolization as sole treatment. Half of the patients showed a durable radiographic response at long-term followup. Later Hosalkar et al. published in 2007 [35] the successful treatment of large GCT of the sacrum by repeated embolization. Nine consecutive patients underwent angiography and SAE at time of diagnosis, followed by repeated embolization every 6 weeks until no new vessels were noted, and then 6 and 18 months thereafter. With this procedure tumor progression was stopped in 7 of 9 cases [35]. We adapted this procedure to treat a surgically inaccessible local recurrence of the lumbar spine (case 6). The patient presented with a local recurrence encasing the abdominal aorta with loss of sensory function at L4 level 9 months after dorsoventral tumor resection. We decided to treat him with serial SAE directly after detection of the recurrence until complete devascularization was achieved (Figure 3). This procedure was repeated after one and six months. After the SAE the neurological impairments completely recovered and the tumor is stable for 19 months now. This is the first description of successful treatment of a spinal GCT with serial SAE which might be adopted for other patients.

In the sacrum most cases were successfully treated by intralesional curettage and bone cement packing. The high recurrence rate of 48% of sacral tumors treated by curettage alone published by Leggon et al. [12] was not confirmed by our data. Possibly our recurrence rate was lower due to the use of cementation whenever possible and SAE in some cases.

The role of EBI as primary or adjunct treatment is still controversially discussed. Despite the relatively high risk of radiation-induced malignancy [36–40], it is still used by many. Leggon et al. published a rate of secondary sarcoma of 11% of patients with pelvic or sacral tumors [12], and Sanjay et al. of 25% in GCT of the pelvis [14]. One of our spine cases (case 3) and one of our sacrum cases (case 9) received EBI for adjuvant treatment of a local recurrence. Three patients with sacral tumors received EBI as an adjunct to initial surgery (cases 12, 15, 16), three as primary treatment (cases 17–19). None of them developed a secondary malignancy so far but as the risk increases with time [41] it might occur at a longer followup. When surgery is an option irradiation should be avoided. Even in inaccessible tumors we by now prefer SAE before considering EBI.

Whereas uncommon for typical GCT a relatively high rate of patients with tumors of the axial skeleton suffer from neurological impairments either at diagnosis or due to therapy. Three of our spine (cases 3, 5, 6) and two of our sacrum patients (cases 16 and 19) had deficits at first presentation. For the spine this rate is in accordance with previous reports of 50 to 70% [19, 22, 25] whereas for the sacrum it seems to be lower. All of our spine patients recovered from their impairments after treatment whereas in the sacrum the deficits were persistent. One spine patient (case 1) developed a loss of sensory function and paresthesias, one sacrum patient (case 7) developed neurological claw toes, another (case 11) a conus/cauda syndrome after surgery. One patient (case 9) developed a footdrop due to progress of a sacral tumor. Thus compared to GCT of the long bones, surgical treatment is accompanied by a high risk of neurological deficits.

Although the recent literature describes positive effects of systemic bisphosphonate administration [42–44], their role in therapy of GCT is still not fully understood. Due to the dismal prognosis of GCT of the spine and sacrum and the relatively rare side effects we nowadays recommend additional bisphosphonates in complicated cases and metastases. Whether there really is an effect has to be answered in the future. A promising agent which is increasingly used in GCT is denosumab, a human monoclonal antibody against RANK ligand, which is able to inhibit osteoclast function. In 2010 Thomas et al. investigated its effect on tumor-cell survival and tumor growth in patients with recurrent or unresectable GCT and found that 86% had a tumor response [45]. Although its effect has to be proven in larger and independent studies, denosumab might be a new treatment option especially in complicated cases.

Although some authors advocate treatment algorithms for spinal [46] and sacral [22] tumors, the decision still has to be made for each case individually. Case presentations such as presented here are the only published references when treating these rare tumors, and are of high importance. In our study as in all other published case series the different treatment regimens were very heterogeneous, ranging from conservative treatments such as SAE or EBI to en bloc spondylectomy. Statistical analysis of different treatment modalities is impossible. However, the published experiences of successful therapies might be transferred to other patients. Whereas spinal tumors should be treated more aggressively intralesional curettage seems to be effective in sacral tumors. Whenever surgery is not possible selective arterial embolization might be considered even for GCT of the spine.

## 5. Conclusion

Compared to the long bones GCT of the axial skeleton has to be considered as a severe disease with a high local recurrence rate. Neurological deficits caused by the tumor itself or its treatment are common. En bloc spondylectomy combined with embolization is the treatment of choice whenever possible. In case of inoperability serial arterial embolization seems to be an alternative not just for tumors of the sacrum but also of the mobile spine.

##  Authors' Contributions

M. Balke: idea of the study, preparation of the paper, and data collection. M. P. Henrichs: followup of patients and collection of literature. G. Gosheger: surgical methods and evaluation of surgical protocols. H. Ahrens: preparation of figures and critical discussion of results. A. Streitbuerger: data collection, followup of patients. M. Koehler: evaluation of radiological data, arterial embolization. V. Bullmann: surgical methods and evaluation of outcome. J. Hardes: supervisor, critical discussion and guidance during writing process, and evaluation of data.

## Figures and Tables

**Table 1a:** (a)

Number	Sex	Age	Site/neuro status	FU	Primary treatment	Rec.	Treatment rec.	Met.	Outcome	
1	M	18 Y	Th6/OK	27 M	Ventral: intral. res., bone graft, ventral stabilization	8 M	Ventral: intral. res. Th6–Th7, bone graft		NED	Loss of sensory function and paresis at Th9 level, footdrop right

2	F	17 Y	Th12 / encasing of left nerve root with sensory disorders	98 M	dorsal: intral. curettage, dorsal instr. Th10 – L2, transection of nerve root Th12 + bone graft, bone cement				NED	

3	F	23 Y	Th10/infiltration of spinal canal with initial paresthesias	83 M	Dorsoventral: intral. partial res. Th10, dorsal instr. Th9–11, bone graft	11 M	Dorsal: intral. partial res. Th9–Th11, decompression of spinal canal, extension of instrumentation Th8–Th12	13 M	SD	Recovery from paresthesias but local progress, destruction of Th7–Th9, encasing of aorta, displacement of cava and heart, pleural/pericardial effusion, bipulmonary met. treated by chemo (4 cycles Ifosfamid, Cisplatin) and EBI 46 Gy over 1 M. last FU constant unresectable met., decrease of local tumor, pregnant

4	F	26 Y	Th11/OK	24 M	Dorsoventral: intral. partial res. Th11, dorsal instr. Th10–Th12, bone graft (rib)	7 M/13 M	Ventral: intral. res., bone cement, laceration of aorta, severe bleeding		D	EBI started after rec., death due to pulmonary failure 13 days after last surgery

5	F	27 Y	L4/infiltration of right psoas/spinal canal, encasing right nerve root with sensory disorders	32 M	Dorsoventral: spondylectomy L4, intral. res. soft tissue component, dorsal instr. L3– L5, titanium cage interposition, bone graft			16 M	NED	Oral clodronate (800 mg 2/d), since resection of bipulmonary met., free of complaints

6	M	30 Y	L4/infiltration of right psoas muscle and spinal canal causing weakness of right quadriceps	45 M	Dorsoventral: intral. spondylectomy L4, intral. res. soft tissue component, dorsal instr. L3–L5, titanium cage interposition, bone graft	9 M	No surgery, serial SAE until complete devascularization 3 times (directly, 1 and 6 M later)		SD	Local rec. encasing aorta with loss of sensory function at L4 level, after serial SAE complete recovery of neurological functions, slight regression of tumor on MRI, since rec. daily oral pain medication and clodronate (800 mg 2/d), able to work full time in office

FU: followup, rec.: recurrence, met.: metastasis, M: male/months, F: female, Y: years, Th: thoracal spine, L: lumbar spine, intral.: intralesional, res.: resection, instr.: instrumentation, EBI: external beam irradiation, preop.: preoperative, SAE: selective arterial embolization, Gy: Gray, NED: no evidence of disease, SD: stable disease, D: dead due to disease.

**Table 1b:** (b)

Number	Sex	Age	Site /neuro status	FU	Primary treatment	Rec.	Treatment rec.	Outcome	
7	F	25 Y	Sacrum/OK	133 M	Intral. curettage, bone cement	11 M	Resection of soft tissue recurrence	NED	Local infection after initial surgery cured by repeated curettage and cementation plus systemic antibiotics, subsequently irritation of left S1 nerve root causing claw toes

8	F	19 Y	Sacrum/OK	124 M	Intral. curettage, bone cement			NED	Local infection after initial surgery cured by repeated curettage and cementation plus systemic antibiotics, final FU free of complaints

9	M	20 Y	sacrum, affection of SI joint/OK	20 M	Intral. curettage, bone cement	12 M	Preop. SAE, partial intral. res.	PD	Local progress 6 M after rec. treated by SAE, another progress after again 6 M treated by EBI 30 Gy over 1 M without effect, at last FU free of pain but progress with incomplete paresis of left foot. Scheduled for denosumab treatment

10	F	20 Y	Sacrum, infiltration of spinal canal/no neurological deficits	62 M	Intral. curettage, bone cement			SD	Stable left-over tumor tissue after partial removal, oral clodronate (800 mg 2/d) over 1 Y, last FU free of complaints

11	F	61 Y	Sacrum/OK	24 M	Intral. curettage, laminectomy S1–S4, bone cement			NED	Conus/cauda syndrome since surgery

12	F	18 Y	Sacrum, crossing of midline/OK	49 M	Intral. partial res. mainly of soft tissue component, chemotherapy (CWS-96 study ifosfamid, vincristine, adriamycin), EBI 50 Gy over 1 M			SD	Free of complaints

13	M	24 Y	Sacrum/OK	88 M	Preop. SAE, ligation of left and right internal iliac vessels and median sacral artery, curettage, bone graft			NED	Local infection after initial surgery cured by repeated curettage and cementation plus systemic antibiotics, final FU free of complaints

14	F	32 Y	Sacrum, infiltration of ilium/OK	15 M	Preop. SAE, curettage, bone cement			NED	Free of complaints
15	M	20 Y	Sacrum, infiltration of spinal canal and spinal cord compression/no neurological deficits	28 M	Preop. SAE, intral. partial curettage, bone cement, EBI with a special particle accelerator at the DKFZ (“german cancer research center” in Heidelberg, Germany) 66 Gy over 1 M			SD	Free of complaints, regression of tumor on MRI

16	M	28 Y	Sacrum, stenosis of spinal canal/ conus/cauda syndrome	51 M	Preop. SAE, partial curettage, bone cement, EBI 60 Gy over 1 M			NED	Persistent rectum/bladder dysfunction, gluteal dysesthesia

17	F	28 Y	Sacrum large/OK	36 M	No surgery, EBI 50 Gy over 1 M	21 M	No surgery, EBI 50 Gy over 1 M	SD	Regression after first EBI but progress after 21 M again treated by EBI + systemic interferon alpha, ongoing oral clodronate (800 mg 2/d) intake since rec.

18	F	29 Y	Sacrum, large, crossing of midline/OK	24 M	No surgery, EBI 55 Gy over 1 M			SD	slight regression of tumor on CT, free of complaints

19	F	56 Y	Sacrum/pain both thighs and buttocks, paresthesias of anal/genital area, buttocks, foot soles, rectum/bladder dysfunction	17 M	No surgery, EBI 50 Gy over 1 M, SAE 3 M later			SD	Significant regression of symptoms since SAE but still paresthesias both foot soles and moderate bladder dysfunction, daily oral pain medication, oral clodronate (800 mg 2/d) since treatment

FU: followup, rec.: recurrence, M: male/months, F: female, Y: years, intral.: intralesional, res.: resection, EBI: external beam irradiation, preop.: preoperative, SAE: selective arterial embolization, Gy: Gray, NED: no evidence of disease, SD: stable disease.
